# Primary Adenotonsillar Tuberculosis in an Immunocompetent Child -A Case Report

**Published:** 2019-09

**Authors:** Chee-Chean Lim, Khairunnisak Misron, Siow-Ping Loong, Yew-Toong Liew, Halimuddin Sawali

**Affiliations:** 1 *Department of Otorhinolaryngology - Head and Neck Surgery, University of Malaya, Kuala Lumpur, Malaysia*; 2 *Department of Otorhinolaryngology - Head and Neck Surgery, * *Queen Elizabeth Hospital, Kota Kinabalu, Sabah, Malaysia.*

**Keywords:** Adenoid, Child, Tonsil, Tuberculosis

## Abstract

**Introduction:**

Primary tuberculosis (TB) of the oropharynx and nasopharynx is an extremely rare form of extra-pulmonary TB in children. Primary tuberculosis occurs more likely secondary to pulmonary TB and is more common in immunocompromised patients.

**Case Report:**

We reported the case of a young male presented with the symptoms of non-specific chronic adenotonsillitis, mild obstructive sleep apnoea, and cervical lymphadenopathy. Subsequently, he underwent adenotonsillectomy and excision of the cervical lymph node with the tissue specimens came back strongly positive for TB. Then, he started using antituberculous medication and recovered well.

**Conclusion:**

The authors would like to highlight this rare clinical entity in which accurate diagnosis is essential for complete treatment.

## Introduction

Currently, the incidence of tuberculosis (TB) is rising, and more new cases have been reported particularly from South East Asia due to the prevalence of acquired immune deficiency syndrome (AIDS), drug resistance, and global migration. Extrapulmonary TB accounts for approximately 25% of overall morbidity(1). TB of the lymph node is the most common extrapulmonary cause with other possible sites, including pleural TB, skeletal TB, TB of the central nervous system, abdominal TB, genitourinary TB, and TB pericarditis. The TB of oropharynx and nasopharynx is rare with a reported incidence of less than 5% (2). In this study, we described a rare case of TB involving the tonsil and adenoid in a child presented with chronic tonsillitis and obstructive sleep apnoea symptoms.

## Case Report

**Fig 1:**
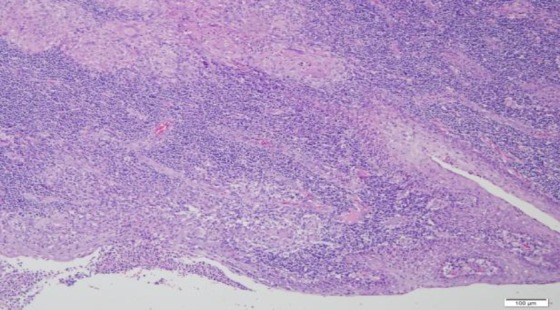
Granulomas are seen here in the tonsil (left upper) with presence of a few multinucleated giant cells, which is seen lined by squamous epithelium in the right lower corner (hematoxylin-eosin; x100)

**Fig 2:**
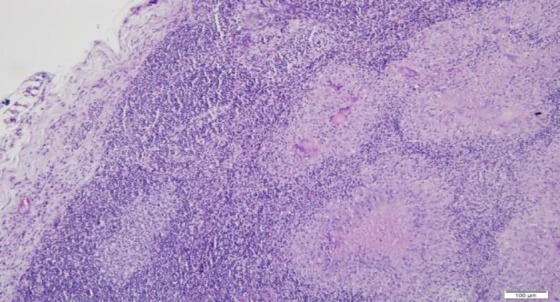
Granulomas also seen in the lymph node (on the right), with a reactive lymphoid follicles seen on the lower left just underneath the capsule (hematoxylin-eosin; x100)

**Fig 3:**
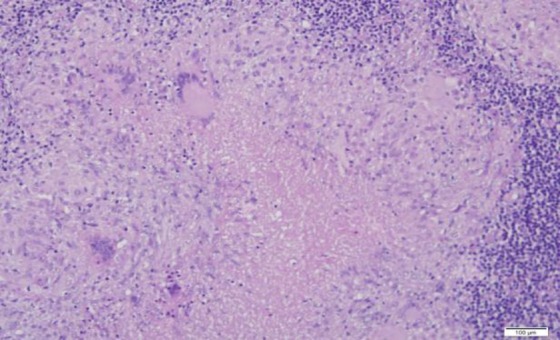
The granulomas consist of central necrosis surrounded by epithelioid histiocytes. Three multinucleated giant cells are seen here (x200).

**Fig 4:**
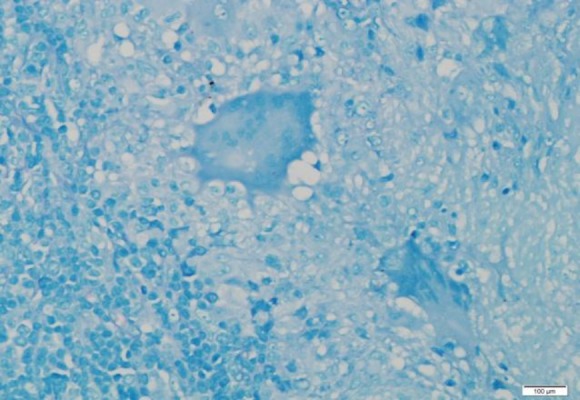
Ziehl-Neelsen stain show presence of an acid-fast bacilli within the multinucleated giant cell (in red) (x400).

A six-year-old boy presented with multiple neck swelling for a month which was increasing gradually in size and number and was painless. Moreover, the child had recurrent sore throat associated with snoring and awakening at night. There was no observed cessation of breathing at night and no chronic cough or hemoptysis. Further history also did not reveal any contact with patients suffering from pulmonary TB. On examination, there were matted lymph nodes measured 4×4 cm palpable on the right side. However, on the left, there were few 2×1 cm palpable lymph nodes. The swellings were firm in nature, non-tender, and mobile. There was symmetrical Grade III hypertrophy of the tonsils with no ulceration. The adenoids were also hypertrophied partially occluding the choana. Otherwise, flexible laryngoscopy and ear examination revealed normal findings. His erythrocyte sedimentation rate was elevated, and Mantoux test reading was 15mm. There were no acid-fast bacilli (AFB) detected from his morning sputum analysis. His chest X-ray was normal. Lactate dehydrogenase test and peripheral blood film were also not significant. The fine needle aspiration cytology of the neck swelling was ordered; however, the results demonstrated reactive cervical lymphadenopathy. Therefore, he underwent excision biopsy of the neck swelling with adenotonsillectomy due to obstructive sleep apnoea symptoms. The histopathological examination of the tonsil, adenoid, and neck node were all reported to contain numerous granuloma (Fig. 1 and Fig.2) composed of epithelioid histiocytes with central necrosis (Fig.3). Moreover, numerous multinucleated giant cells were detected by the Ziehl-Neelsen stain for acid bacilli that were positive (Fig.4). The child had postoperative bleeding 18 days post-tonsillectomy. He had to undergo an examination under anesthesia with hemostasis to stop bleeding. Subsequently, the case was given a course of anti-tuberculous drug and recovered completely. 

## Discussion

The isolated TB of the tonsil and adenoid is rare, especially in an immunocompetent child. The main cause is the ingestion of unpasteurized milk containing Mycobacterium Bovis. Tonsillar TB is divided into primary TB of tonsil in which it occurs without pulmonary TB. However, the secondary TB of tonsil occurs with pulmonary TB containing positive sputum smear causing the inoculation of tubercle bacilli in Waldeyer’s ring. 

Miller concluded that tonsillar TB decreased in the advent of pasteurized milk, and the secondary form is more common in present day (3). The route of spread may be due to the inhalation of tubercle bacilli which harbors in Waldeyer’s ring or due to hematogenous spread. Poor dental hygiene, leukoplakia, periodontitis, and post dental extraction are also known predisposing factors of primary tonsillar TB (4). Other studies have also reported an increased incidence in immunocompromised host due to retroviral disease, alcoholism, and substance abuse (5).

Tonsil is one of the lymphatic structures with constant easy contact to infected sputum; however, the incidence of TB tonsil is low due to the inhibitory effect and antiseptic action of saliva. The presence of saprophytes in the oral cavity also antagonizes bacterial invasions through the striated musculature. The rather thick protective stratified squamous epithelium encasing the tonsil also resist the colonization of Mycobacterium TB (6). The clinical presentations of tonsillar TB are non-specific. Patients may present with a sore throat, odynophagia, dysphagia, enlarged tonsils, and cervical lymphadenopathy with or without constitutional symptoms. Asymmetric tonsillar enlargement, obliteration of crypts with white patches, and enlarged jugulodigastric nodes may point towards tonsillar TB; however, these may mimic tonsillar malignancies, which are more common in the elderly. Other differential diagnoses of TB tonsil include aphthous ulcer, actinomycosis, hematological disorders, traumatic ulcer, Wegener’s granulomatosis, and midline granuloma (7). Therefore, clinical suspicion should prompt TB workout, especially in regions with a high incidence of TB.

The Mantoux test is one of the supportive methods in the diagnosis of tonsillar TB; nonetheless, it has limited diagnostic value. The results of the aforementioned test can be complicated by latent TB infection and previous Bacille Calmette-Guerin vaccination. Reduced host response due to HIV infection, immunocompromised patients, and poor nutrition status can also lead to false negative results. 

Definitive diagnosis warrants tissue biopsy obtained by punch biopsy or after tonsillectomy to demonstrate the presence of epithelioid granuloma with caseation necrosis and Langerhans giant cells with or without the presence of AFB from the Ziehl-Neelsen staining (8). Mycobacterium culture of the sampled tissue can also be performed using nucleic acid amplification tests, such as polymerase chain reaction, to improve sensitivity. It can detect as few as 10 mycobacteria, especially in extrapulmonary TB, which is a paucibacillary TB disease (9).

Chest radiography and sputum AFB should be conducted to exclude concurrent pulmonary TB warranting isolation. Despite normal chest X-ray findings, positive sputum cultures may be detected in some TB patients with the option of bronchoscopic evaluation or sputum induction with hypertonic saline nebulization to assist diagnostic sensitivity (10). The HIV status of the patient diagnosed with TB tonsil should also be screened as it is more commonly associated with poor cell-mediated immunity (5).

## Conclusion

The TB of the tonsil and adenoid should be suspected in the presence of enlarged cervical lymphadenopathy, especially with suspicious tonsils even in a child who is immunocompetent. This is particularly true in regions where TB is endemic due to its potentially life-threatening consequences. Presenting symptoms and signs can be non-specific, and definitive tissue biopsy is imperative for accurate diagnosing and achieving complete cure.
